# Experimental and Numerical Assessment of Fibre Bridging Toughening Effects on the Compressive Behaviour of Delaminated Composite Plates

**DOI:** 10.3390/polym12030554

**Published:** 2020-03-03

**Authors:** Aniello Riccio, Angela Russo, Andrea Sellitto, Cinzia Toscano, Davide Alfano, Mauro Zarrelli

**Affiliations:** 1 Department of Engineering, University of Campania “L. Vanvitelli”-via Roma, 81031 Aversa, Italy; aniello.riccio@unicampania.it (A.R.); andrea.sellitto@unicampania.it (A.S.); 2 CIRA Italian Aerospace Research Centre, Via Maiorise s/n, 81043 Capua, Italy; c.toscano@cira.it (C.T.); d.alfano@cira.it (D.A.); 3 Institute of Polymers, Composites and Biomaterials, CNR–Research National Council of Italy, Granatello 80055 Portici, Naples, Italy; mauro.zarrelli@cnr.it

**Keywords:** delamination, fibre bridging, crack propagation, snap-through buckling, compressive tests

## Abstract

Increasing the Mode I inter-laminar fracture toughness of composite laminates can contribute to slowing down delamination growth phenomena, which can be considered one of the most critical damage mechanisms in composite structures. Actually, the Mode I interlaminar fracture toughness (*G_Ic_*) in fibre-reinforced composite materials has been found to considerably increase with the crack length when the fibre bridging phenomenon takes place. Hence, in this paper, the fibre bridging phenomenon has been considered as a natural toughening mechanism able to replace embedded metallic or composite reinforcements, currently used to increase tolerance to inter-laminar damage. An experimental/numerical study on the influence of delamination growth on the compressive behaviour of fibre-reinforced composites characterised by high sensitivity to the fibre bridging phenomenon has been performed. Coupons, made of material systems characterised by a variable toughness related to a high sensitivity to the fibre bridging phenomenon and containing artificial through-the-width delaminations, were subjected to a compressive mechanical test and compared to coupons made of standard material system with constant toughness. Out-of-plane displacements and strains were monitored during the compression test by means of strain gauges and digital image correlation to assess the influence of fibre bridging on delamination buckling, delamination growth and on the global buckling of the specimens, including buckling shape changes. Experimental data were combined with a numerical study, performed by means of a virtual crack closure technique based procedure, named SMart Time XB – Fibre Bridging (SMXB-FB), able to mimic the crack bridging effect on the toughness properties of the material system. The combination of numerical results and experimental data has allowed the deformations and the buckling shape changes to be correlated to the onset and evolution of damage and, hence, contributes to improving the knowledge on the interaction of the failure mechanisms in the investigated composite specimens.

## 1. Introduction

Fibre bridging is a toughening mechanism which is able to delay delamination growth in composite structures [1,2,3]. Indeed, delaminations are among the most dangerous failure mechanisms for composite material structures, due to their frequent unstable growth, which can lead to the sudden and premature collapse of structures [4,5,6,7,8]. Actually, fracture toughness is a non-isotropic matrix property able to slow down damage progression in the weaker (out-of-plane) direction of fibre-reinforced composite laminates. Hence, improving this material property becomes mandatory to widen the possible applications of fibre-reinforced composite laminates.

To improve the fracture toughness, toughening resins and thickness reinforcements (stitching, pinning or orthogonal weaving) are generally adopted [9,10,11,12,13]. Such reinforcements, even if able to slow down and sometimes arrest the delamination advance, can strongly reduce the in-plane mechanical properties of the fibre-reinforced composites. Indeed, in the stitching process a needle is used to pierce the laminate, in order to insert a high tensile strength yarn. Literature study [14] demonstrates that stitching can cause breakage, kink and spread of the fibres. Furthermore, the formation of resin-rich regions, porosity and resin cracks can occur. Likewise, the presence of the z-pins creates resin pockets and skew of the fibres, leading to degradation of the in-plane strength of the composite [15]. Another highly effective method to improve the fracture toughness is the addition of nanoparticles in the polymeric matrix [16,17,18,19,20,21], which improves also tensile strength, stiffness and thermal properties. On the other hand, some disadvantages are associated with nanoparticles, such as viscosity increase, dispersion problems and sedimentation [22]. Hence, attention should be paid to alternative toughening approaches based on the modification of the manufacturing process of composites that are able to tune the sensitivity to natural toughening phenomena, such as the fibre bridging.

In order to experimentally assess the effects of toughness variations on the mechanical behaviour of composite components, Non-Destructive Testing (NDT) methods can be used, which, replacing the more uncertain “destructive sample testing”, allow the evolution of inter-laminar damage under loading conditions to be evaluated. Among others, Digital Image Correlation (DIC), which is a technique based on the acquisition of surface digital images at different loading stages, allows the strain and the displacement distributions all over the specimen to be evaluated. The analysis and comparison of the acquired images can give an idea of the damage evolution in the inspected structures [23,24,25,26]. The main advantage of the DIC is the ease of use, since significantly less equipment is needed for DIC inspections compared to other NDT techniques, such as the ones based on ultrasound [27,28] and thermography [29,30].

In this paper, a numerical/experimental study, on the compressive behaviour of delaminated composite plates, is introduced. The main aim is to investigate the effects of modifications to the manufacturing process of composites for tuning the sensitivity to fibre bridging. Two material systems have been considered: the former is a material highly sensitive to fibre bridging characterised by a toughness increase with crack length; the latter is a toughened material with constant toughness. Epoxy resin/carbon fibre coupons, characterized by an artificial through-the-width delamination, have been experimentally tested under compression to assess their inter-laminar damage behaviour. The specimens have been equipped with strain gauges to investigate the delamination buckling and growth, as a result of the compressive load. Moreover, a DIC system has been employed for the monitoring of displacements and deformation fields during the test.

A numerical study has also been carried out and the results have been used, together with the experimental data, to provide an interpretation of the effects of the Mode I fracture toughness on delamination phenomena. The adopted numerical tool, validated in [31], is an innovative and robust Virtual Crack Closure Technique (VCCT)-based procedure, named SMXB-FB, able to simulate the interlaminar damage in composite material structure, taking into account the bridging of the fibres, as well as the resistance curve behaviour (variation of toughness with crack length).

In Section 2, the manufacturing of the investigated plates is analysed in detail and information is provided on the experimental procedures behind the compressive mechanical tests. In Section 3, the SMXB-FB numerical tool and the finite element model are introduced. Finally, in Section 4, the experimental data and the numerical results are presented, compared and discussed in order to improve the knowledge about the interactions between fibre bridging and buckling behaviour of the investigated plates.

## 2. Specimen Manufacturing and Experiments Details

**Figure 1:**
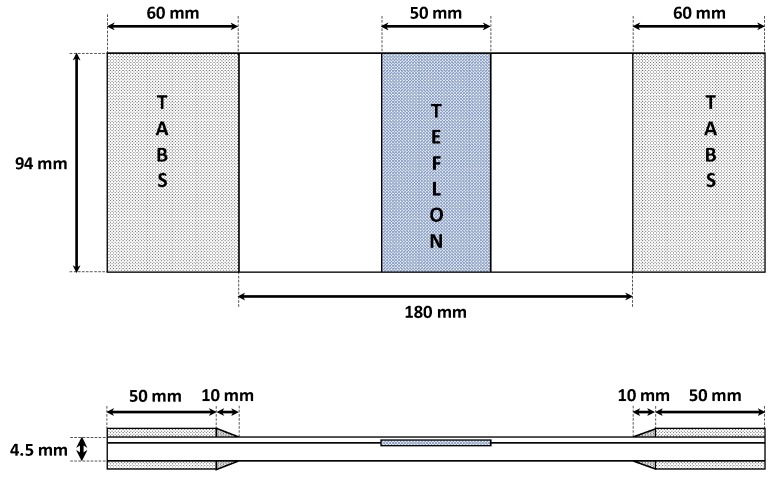
Geometrical description of the specimens.

Epoxy resin/carbon fibre specimens with a 50-mm wide trough-the-width artificial delamination were manufactured according to the geometrical description given in Figure 1. Coupons are made of 24 plies (each ply is 0.1875 mm thick) with a stacking sequence of [0,90]_6s_. A rectangular Teflon film was inserted between the 4th and 5th ply, as shown in Figure 1, to create an artificial delamination. Tabs on both sides of the samples were placed to allow a distribution as uniform as possible of the load from the test machine to the sample.

Two different curing processes have been used to manufacture the epoxy resin/carbon fibre plates. As a result, two sets of coupons have been obtained with the same mechanical properties but different Mode I fracture toughness:

- set of coupons characterised by a constant *G_Ic_* of 510 J/m^2^ (toughened material);
- set of coupons characterized by a variable *G_Ic_*, from 243 J/m^2^ to 456 J/m^2^ depending on delamination size (material sensitive to fibre bridging).

**Figure 2:**
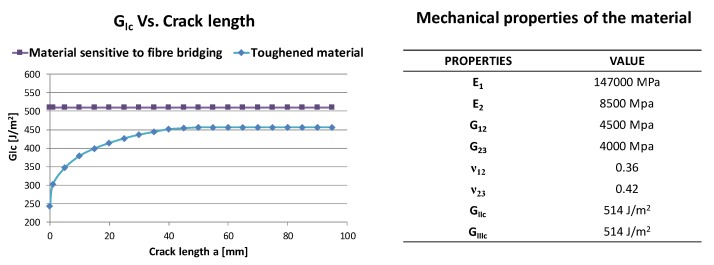
Resistance curves and mechanical material properties.

The resistance curves for the two sets of coupons, representing the *G_Ic_* as a function of the crack length *a*, are reported in Figure 2, together with the other mechanical properties.

**Figure 3:**
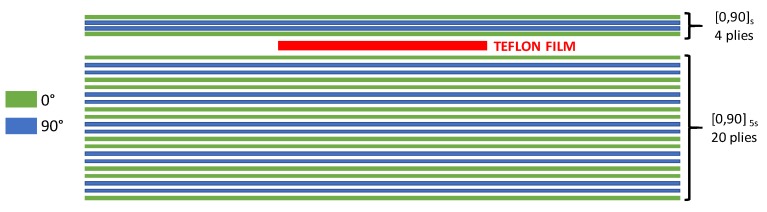
Specimen stacking sequence and delamination position along the thickness.

Three samples were cut from each plate, resulting in three specimens made of toughened material and three specimens made of material sensitive to fibre bridging. In Figure 3, the stacking sequence and the position of delamination along the thickness, between the fourth and fifth ply, are introduced.

The tested coupons were labelled according to their fracture toughness characteristics, i.e., toughened material (TOUGH) and material sensitive to fibre bridging (MFB), as listed in Table 1, where the tests matrix is reported.

**Figure 4:**
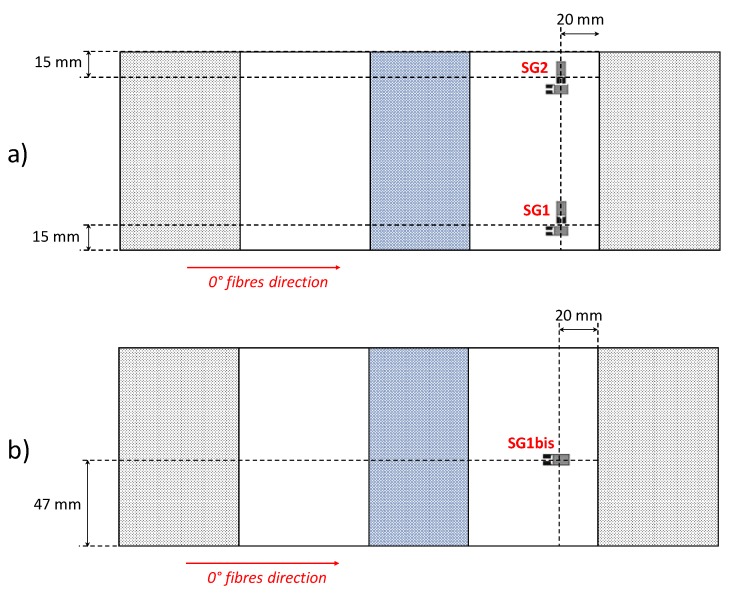
Strain gauges position in (**a**) TOUGH#1, TOUGH#2, MFB#1 and MFB#2 specimens; (**b**) TOUGH#3 and MFB#3 specimens.

According to Table 1, the TOUGH#1, TOUGH#2, MFB#1 and MFB#2 specimens were equipped with two strain gauge rosettes (SG1 and SG2), located, as shown in Figure 4a, on the thicker sub-laminate side, to measure the deformations in the 0° and 90° fibre directions. The remaining samples, TOUGH#3 and MFB#3, were prepared for the DIC by painting in white and by randomly sprinkling with black pigments the thinner sub-laminate surfaces. These two last specimens were also equipped with one 0° fibre direction strain gauge (SG1bis), placed, as shown in Figure 4b, on their thicker side.

**Figure 5:**
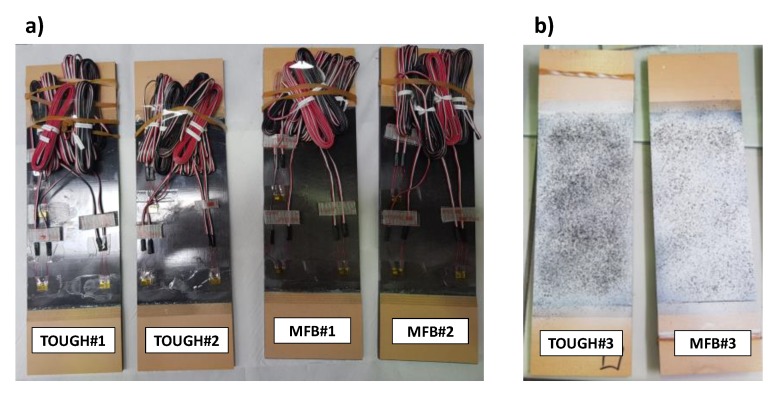
Samples preparation for strain monitoring (**a**) and DIC (**b**).

Images of the specimens are shown in Figure 5. Indeed, in Figure 5a the specimen prepared for strain monitoring with strain gauges is shown, while in Figure 5b the specimen prepared for DIC is presented.

**Figure 6:**
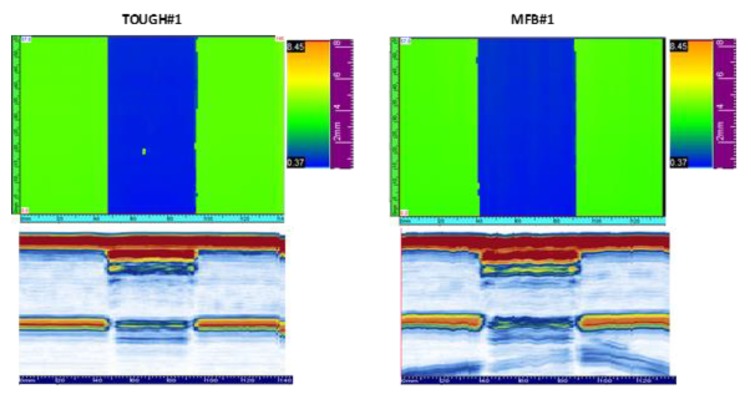
Ultrasonic inspection.

Before the compression tests, the samples were checked for manufacturing defects using ultrasonic inspections, performed by means of an ultrasonic device with an array of 64 sensors. No relevant defects have been found apart from some small voids at the edges of the Teflon film, probably due to the roughness of the Teflon film self. Figure 6 shows the C-scans and the B-scans of the TOUGH#1 and MFB#1 samples, as an example of the performed non-destructive ultrasonic checks.

**Figure 7:**
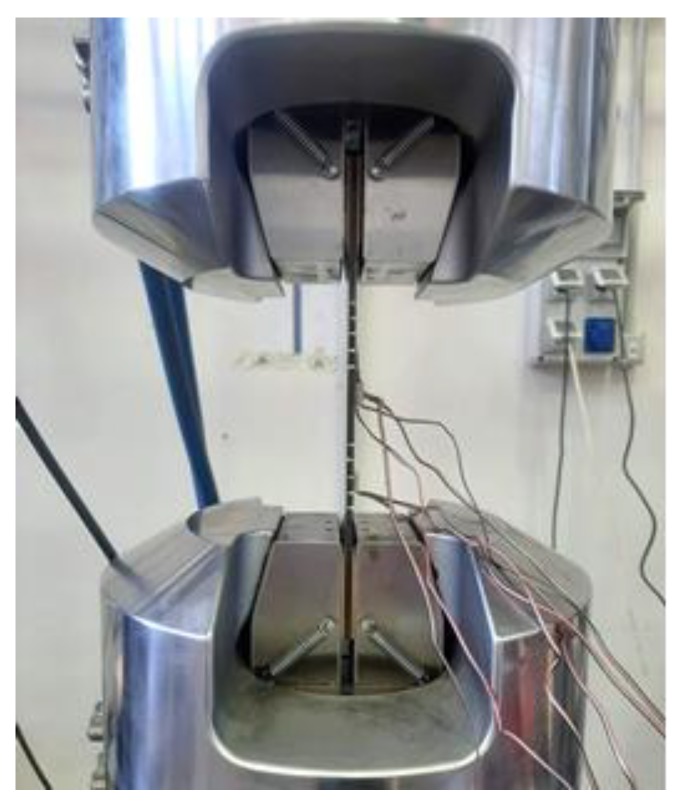
Test rig.

A hydraulic testing machine was used to perform compressive mechanical tests. The tabs of the specimens were clamped in the hydraulic grip and a controlled compressive displacement was applied with a rate of 0.5 mm/min. A picture of the specimen mounted in the testing rig is shown in Figure 7. After the installation of each specimen, the strain gauges were linked to the data acquisition systems and calibrated. Consequently, the specimens were loaded with a small force before the test initiation in order to check the strain gauge measurements and verify the correct alignment and the proper introduction of the compressive load.

According to the test matrix of Table 1, DIC inspections were performed on TOUGH#3 and MFB#3 samples. DIC is a no-contact measurement technique based on numerical processing of digital images for the analysis of displacement and deformation fields. Through two cameras, several images are acquired, as well as an initial image representing the body in the reference condition, which is typically the undeformed configuration. The subsequent data analysis operations compare the reference image with those acquired with the body in the different deformed conditions, in order to calculate the relative displacements and deformations. In more detail, the area of interest is divided by the processing software into small square regions, named a subset. In each subset, the displacement of the central point is calculated for different time instants.

**Figure 8:**
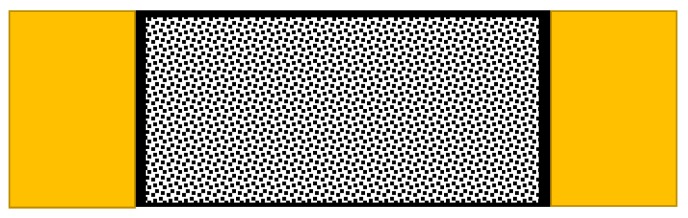
Speckle pattern in Digital Image Correlation (DIC).

Since calculations depend on the subset correspondence within the acquired images, it is clear that the choice of suitable patterns becomes of main relevance. The special pattern used for DIC analysis is called a speckle pattern, which is typically composed of dark speckles of uniform size, distributed on a white background, to maximize contrast, as schematically shown in Figure 8.

Proper correlation criteria have been defined to find the correspondence between each subset in the reference image and in the images acquired during the deformed states (cross correlation criteria and sum-squared differences-based criteria [32,33]).

**Figure 9:**
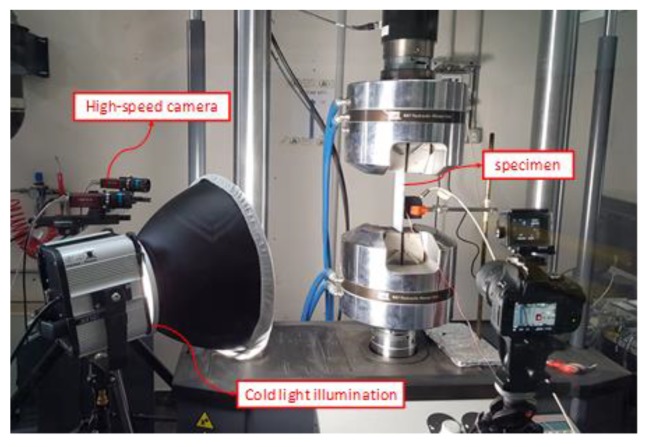
Digital Image Correlation rig.

In Figure 9, the DIC instrumentation used during the tests is shown. The digital images were acquired every 3.5 s for the entire duration of the tests.

## 3. Numerical Procedure Description and Finite Elements Model Definition

The experimental research activity, described in the previous section, was numerically simulated with the aim to investigate the damage mechanisms onset, evolution and interaction when delamination grows under compression, depending on the different interlaminar toughness conditions. The robust numerical tool SMXB FB, validated in [33], was used to mimic the compressive behaviour of the abovementioned delaminated composite coupons taking into account the fibre bridging effects.

### 3.1. SMXB FB Numerical Tool Description

The delamination growth phenomenon is usually simulated in commercial Finite Elements Method (FEM) codes by connecting the propagation region nodes’ couple with appropriate contact elements and by defining a propagation criterion able to “kill” such interface elements when proper conditions on the Strain Energy Release Rate (SERR) are met. This method is labelled as Fail Release (FR) approach. The SERR can be calculated by using the Virtual Crack Closure Technique (VCCT) equations, described in detail in [34]. In particular, the VCCT equation for the SERR calculation in the case of 4-noded solid elements is reported in Equation (1).

(1) $$G_{j}=\frac{F_{j}\Delta u_{j}}{2\Delta A}$$

where the subscript j identifies the opening (I), sliding (II) and tearing (III) fracture mode, Fj the force at the crack tip, Δuj the opening displacement and ΔA the delaminated area.

The SMXB-FB numerical tool, implemented in the framework of the ANSYS^®^ FEM software [35], is based on the VCCT-FR approach and employs the linear power law, in Equation (2), as the delamination growth criterion.

(2) $$E_{d}=\frac{G_{I}}{G_{Ic}}+\frac{G_{II}}{G_{IIc}}+\frac{G_{III}}{G_{IIIc}}\geq 1$$

According to Equation (2), the SERR values (Gj with j = I, II, III) are compared with the corresponding critical values (Gjc with j = I, II, III), which are experimentally determined [36,37,38].

The standard VCCT-FR approach usually underestimates or overestimates the delaminated area, due to mesh size and time step dependence, as demonstrated in [39]. The SMXB-FB procedure, is capable of overcoming these dependencies and providing a correct evaluation of the delaminated area as a function of the applied load.

According to Equation (2), when the condition Ed > 1 is verified for a certain load step size, the commercial VCCT-based codes release the constraint between the pair of nodes and define the delaminated zone (ANUM), which depends on the element size (ΔAe). In such a way, an over/underestimation of the damaged area can be achieved. The VCCT-based SMXB-FB numerical procedure iteratively changes the load step size, in order to find the value of applied load for which the nominal released area (AES) exactly corresponds, when the growth criterion Ed = 1 is satisfied, to the numerically computed damaged area (ANUM). This principle is clarified in Equation (3), where N is the number of nodes couples characterized by Ed = 1 and

(3) $$A_{ES}=\sum_{i=1}^{N}(\sum_{j=1}^{3}\frac{F_{ji}\Delta u_{ji}}{2G_{jc}})≅\sum_{i=1}^{N}\Delta A_{i}^{D}=f\cdot \Delta A_{e}=A_{NUM}$$

Additionally, the SMXB-FB procedure is capable of correctly defining the local coordinates system at the delamination front for the computation of SEER components, even for irregular delamination front shapes.

In the SMXB-FB numerical methodology, the ability to overcome the mesh and time step dependences is combined with the capability of taking into account the fibre bridging phenomenon by considering the materials’ resistance curves. Indeed, the growth criterion in Equation (2) has been rewritten as reported in Equation (4) (where a is the crack length).

(4) $$E_{d}=\frac{G_{I}}{G_{Ic}(a)}+\frac{G_{II}}{G_{IIc}}+\frac{G_{III}}{G_{IIIc}}\geq 1$$

**Figure 10:**
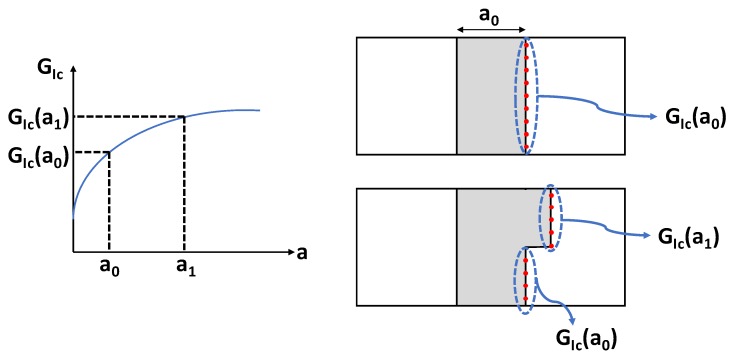
Description of the Fibre Bridging (FB) modulus operation based on the R-curve.

According to Equation (3), when the equivalence is satisfied, the constrains between the nodes are released and the delamination front shape is adapted. As the crack advances, the Fibre Bridging (FB) modulus of the SMXB-FB numerical tool can assign to each node of the new delamination front, resulting from the propagation, a new value of GIc, according to the resistance curve. The FB modulus operating principles are schematically described in Figure 10.

The above-mentioned features, such as time step and mesh independence and the ability to mimic the bridging of the fibres, make the SMXB-FB numerical tool a robust and innovative procedure able to realistically simulate the interlaminar damage evolution in composite structures.

### 3.2. Finite Elements Model

**Figure 11:**
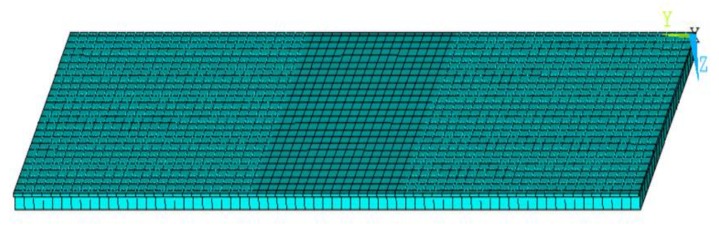
Finite elements model.

**Figure 12:**
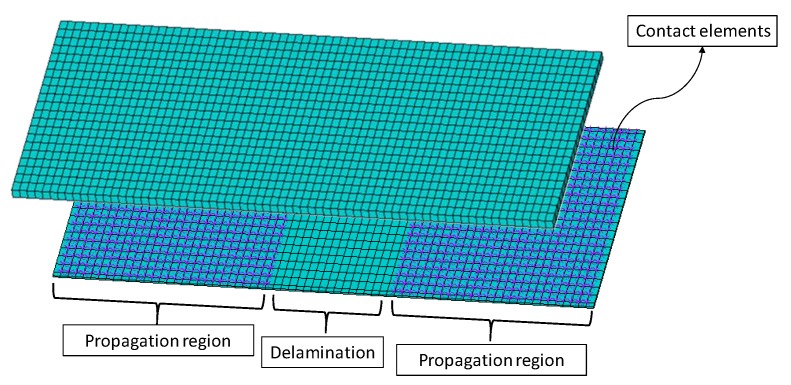
Finite elements model: contact elements.

The finite element model was created according to the tested coupons geometry, shown in Figure 1, and discretized with solid ANSYS^®^ layered elements, as shown in Figure 11. In order to model the region of possible delamination propagation, the couples of nodes, belonging to the two considered sublaminates (see Figure 3) were connected by means of contact elements (see Figure 12). Such contact elements are released depending on whether the propagation criterion is satisfied; this feature is called “birth and death option”.

With the aim to simulate the experimental compressive tests boundary conditions, the panel was clamped on one edge, while compressive displacement was applied to the other edge. The tabs regions were neglected in the FEM model.

## 4. Numerical Results/Experimental Data Comparisons and Discussion

In this section, the experimental strain gauges reading and the DIC images and measurements are compared and integrated with the SMXB-FB numerical results, in terms of strains, out-of-plane displacements and delaminated area, as a function of the compressive load in order to better understand the influence of fracture toughness on the compressive behaviour for the analysed composite plates.

**Figure 13:**
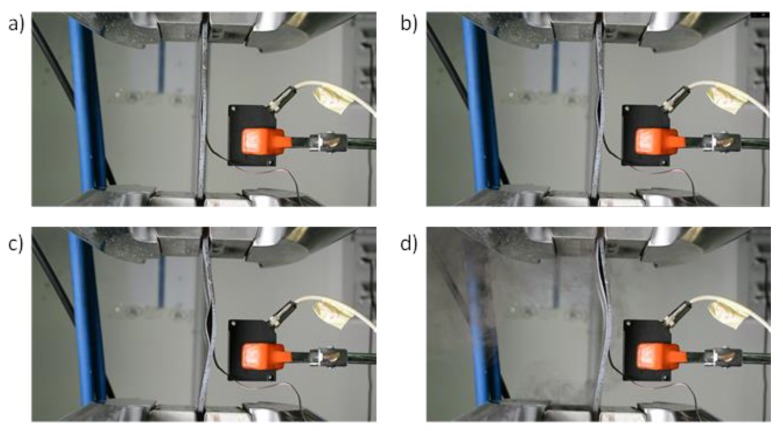
Toughened specimen deformed shape at (**a**) delamination onset, (**b**,**c**) intermediate delamination state and (**d**) complete delamination.

**Figure 14:**
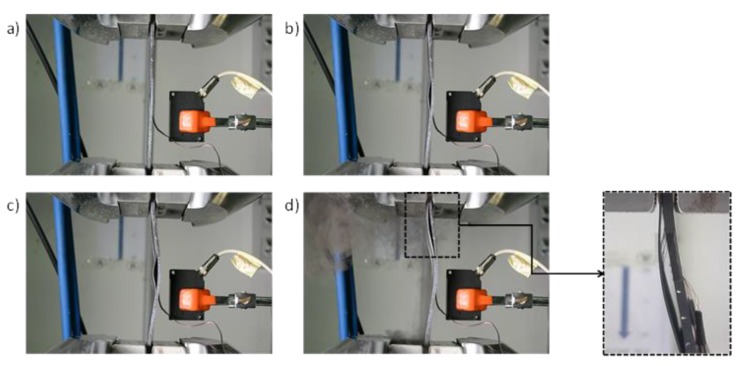
Specimen sensitive to fibre bridging deformed shapes at (**a**) delamination onset, (**b**,**c**) intermediate delamination state and (**d**) complete delamination.

All the tested specimens showed a similar qualitative behaviour when subjected to compressive load. In order to outline the main phases during the compression tests, as an example, pictures taken during the tests are shown in Figure 13 and Figure 14, respectively, for a toughened sample and for a sample sensitive to fibre bridging.

In Figure 13a and Figure 14a the local delamination buckling onset can be seen, while in Figure 13b and Figure 14b the delamination growth onset can be noticed. Figure 13c and Figure 14c clearly show the transition to delamination buckling in the first global buckling shape, which develops in the opposite out-of-plane direction with respect to the delamination buckling. Finally, Figure 13d and Figure 14d show the transition to the second buckling shape (snap-through buckling phenomenon [40,41]) caused by a sudden delamination growth.

The fibre bridging, occurring in the coupons sensitive to fibre bridging, can be seen in Figure 14d (zoomed view).

**Figure 15:**
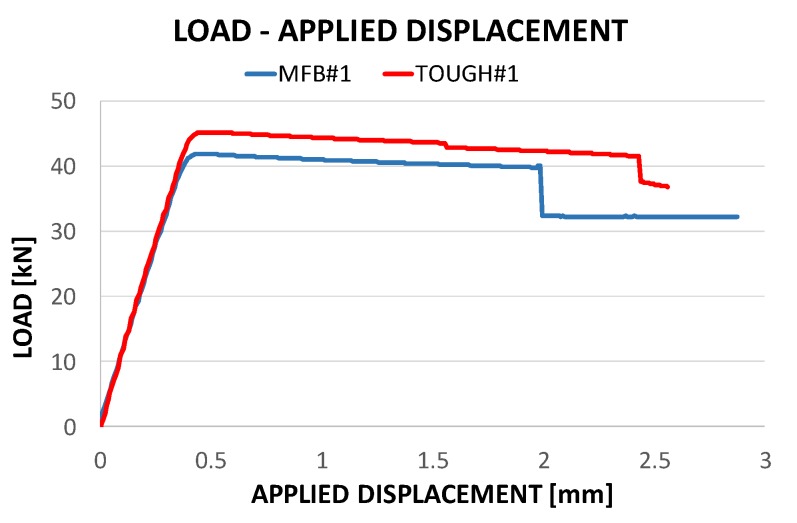
Compressive load vs applied displacements.

Figure 15 introduces the experimental global compressive behaviour of the coupons in terms of load vs displacements curves. Indeed, in Figure 15 a change of slope, highlighting the global buckling phenomenon, can be observed at about 0.5 mm of applied displacement, corresponding to a compressive load of 45.2 kN in the case of toughened material and 41.2 kN in the case of material sensitive to fibre bridging. Moreover, the complete debonding state and the snap-through buckling phenomenon can be appreciated at 2.5 mm (40.4 kN) and 2 mm (39.3 kN) of applied displacement, respectively, in the case of toughened material and in the case of material sensitive to fibre bridging.

In Table 2, a summary of the loads and strains at local delamination buckling, global buckling and snap-through is provided.

From Table 2, it is clear that, as seen in the previous figure, the global buckling and the snap-through buckling are influenced by the fracture toughness characteristics of the material, while, since delamination growth has not yet started, the delamination buckling is, obviously, not influenced by the fracture toughness.

**Figure 16:**
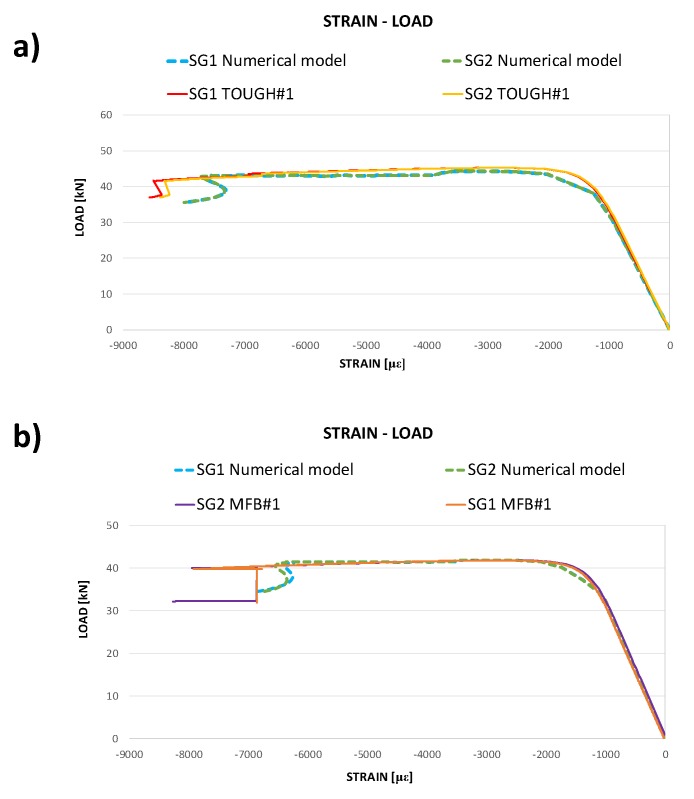
Compressive load as a function of the measured and computed strains in the loading direction: toughened material (**a**) and material sensitive to fibre bridging (**b**).

In Figure 16, the numerical strains in the loading direction as a function of the compressive load are compared to the experimental strain gauge measurements for the toughened specimens and the specimens sensitive to fibre bridging.

According to Figure 16, for both the analysed materials, the bending stiffness and the maximum achieved load are excellently predicted by the SMXB-FB routine. In Figure 16, it is also possible to notice that the snap-through phenomenon is delayed in the case of toughened material and this effect is well predicted by the numerical tool used for the simulations. Indeed, the lower toughness of material sensitive to fibre bridging at delamination growth initiation causes an acceleration of the delamination growth phenomenon with consequent earlier conditions of elastic instability leading to the snap-through event, if compared to the toughened material.

However, the ultimate strains values are slightly underestimated by the numerical code for both the material configurations. Indeed, with the displacement control method, which has been used in this work, the performed static analyses can provide incomplete or misguided information about the stability of the sample in the presence of dynamic phenomena, such as snap-through buckling. Anyway, even in the presence of dynamic snap-through buckling, the trend of numerical results is in excellent agreement with that of experimental outputs, mainly due to the almost quasi-static behaviour of the sample up to the snap-through buckling.

**Figure 17:**
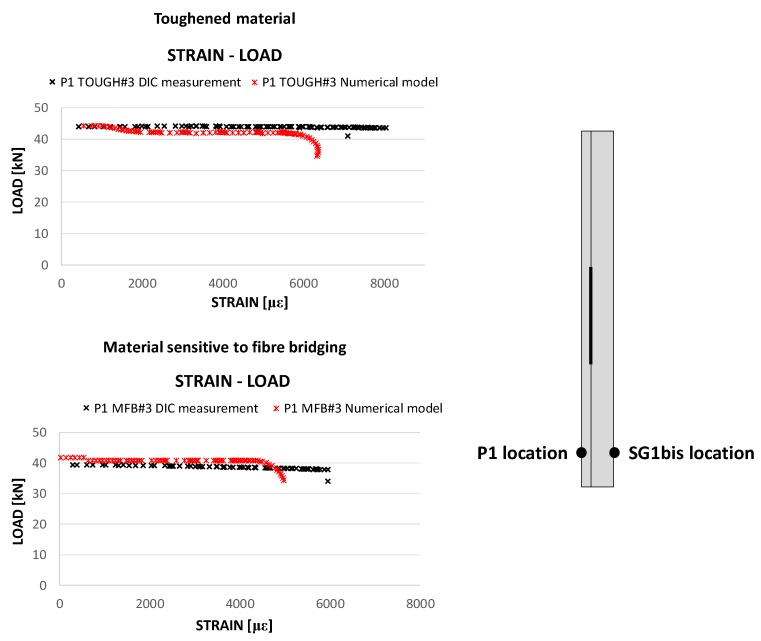
Compressive load as a function of the DIC measured strains: comparison with the numerical results.

Such a discrepancy in the ultimate deformation values is also pointed out in Figure 17, where the strains measured by means of the DIC system at the P1 location (shown in Figure 17) are compared with the numerical strains at the same location. Indeed, at higher strain values, misalignment between numerical and experimental results can be seen because of the presence of dynamic phenomena, which were neglected in the numerical analyses.

**Figure 18:**
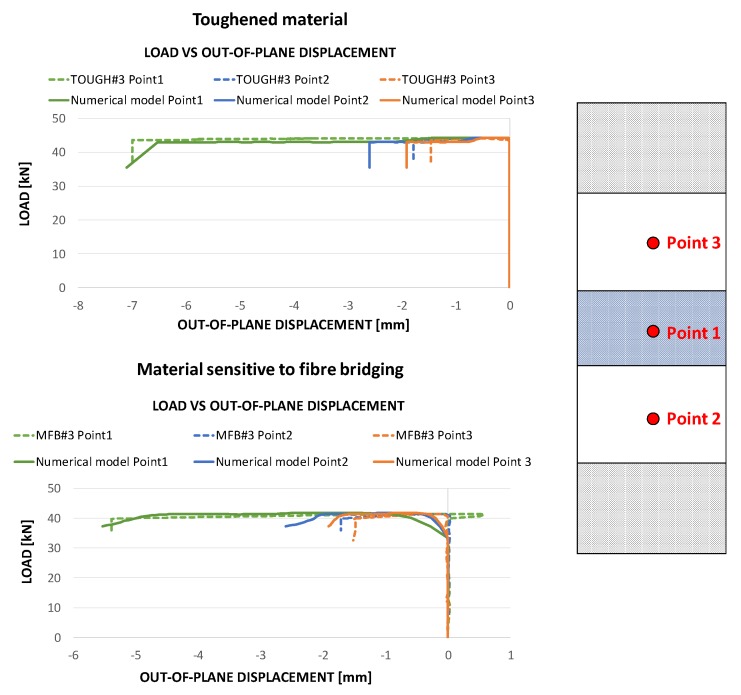
Compressive load as a function of the DIC measured out-of-plane displacements: comparison between experimental data and numerical results.

In Figure 18, the load vs out-of-plane displacements curve, experimentally obtained by the DIC system, for the toughened material and material sensitive to fibre bridging, are compared to the numerical results. According to Figure 18, the out-of-plane displacement at the central location (Point 1) is predicted with excellent accuracy, while the experimentally determined out-of-plane displacements at Point 2 and Point 3 locations are slightly overestimated by the numerical tool. Actually, as seen from the deformed shapes, the dynamic effects due to the buckling shape change are localised at Point 2 and Point 3.

**Figure 19:**
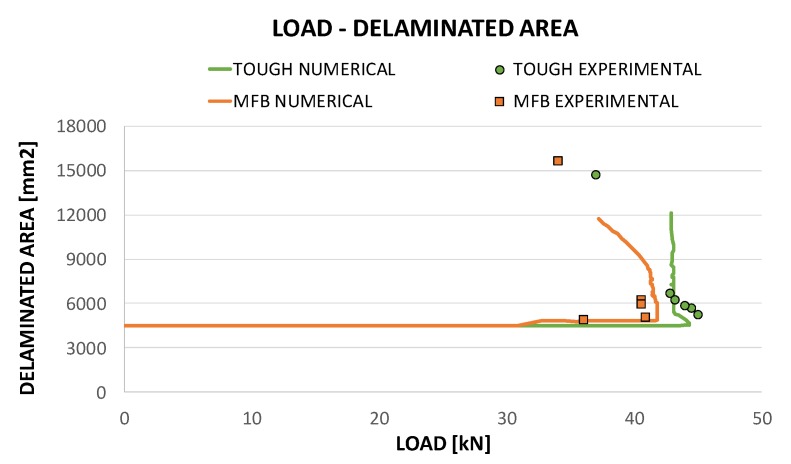
Delaminated area as a function of the compressive load: comparison between experimental data and numerical results.

The growth of the delaminated area was analysed for the two material configurations, both experimentally and numerically during the loading process, providing interesting indications of the GIc influence on the delamination evolution. Numerical results and experimental data in terms of delamination size as a function of the compressive load are presented, compared and integrated in Figure 19, for the two analysed material configurations.

Figure 19 shows that, for both the analysed material configurations, the numerical predictions give a valuable contribution to the clarification of experimental data. Indeed, the trends of the delaminated area vs compressive load point out that, for the material sensitive to fibre bridging, the delamination growth is anticipated (32 kN) with respect to the toughened configuration (43 kN). For the material sensitive to fibre bridging configurations, after a stable delamination opening, an unstable propagation occurs, leading to the snap-through phenomenon. On the contrary, the toughened material is characterised by a highly unstable delamination growth since delamination growth initiation occurs very close to the global buckling. As already pointed out, the numerical tool adopted for the analyses is not able to account for the dynamic effects, hence it is not able to give a prediction of the delamination growth beyond the snap-through phenomenon.

**Figure 20:**
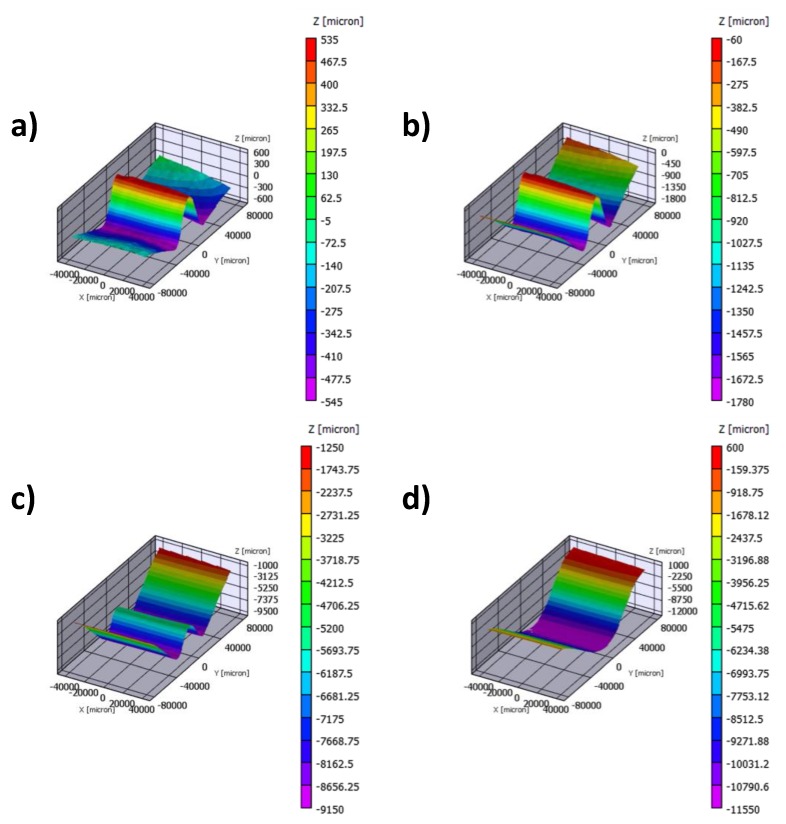
DIC out-of-plane displacement contour plot (MFB#3 sample) at (**a**) delamination buckling onset, (**b**) delamination growth initiation, (**c**) global buckling and (**d**) snap-trough phenomenon.

As already mentioned, digital image correlation inspections were performed on two of the tested samples (TOUGH#3 and MFB#3). Figure 20 shows the out-of-plane displacement contour plot obtained by means of the DIC technique, as an example, for the case MFB#3.

As for Figure 13 and Figure 14, in Figure 20a the local delamination buckling onset can be seen, while in Figure 20b the delamination growth onset can be pointed out. Figure 20c clearly shows the transition from delamination buckling to the first global buckling shape which develops in the opposite out-of-plane direction with respect to the delamination buckling. Finally, Figure 20d shows the transition to the second buckling shape caused by a sudden and unstable delamination growth phenomenon.

**Figure 21:**
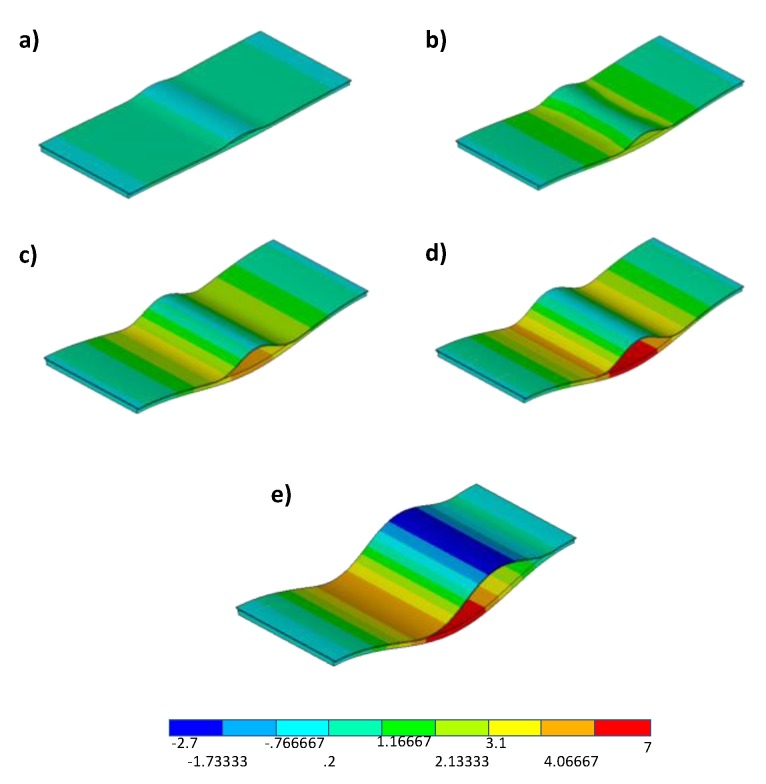
Numerical out-of-plane displacement contour plot for toughened material (units in mm) at (**a**) 0.3 mm applied displacement, (**b**) 0.4 mm applied displacement, (**c**) 0.55625 mm applied displacement, (**d**) 0.73125 mm applied displacement, and (**e**) 1.18 mm applied displacement.

**Figure 22:**
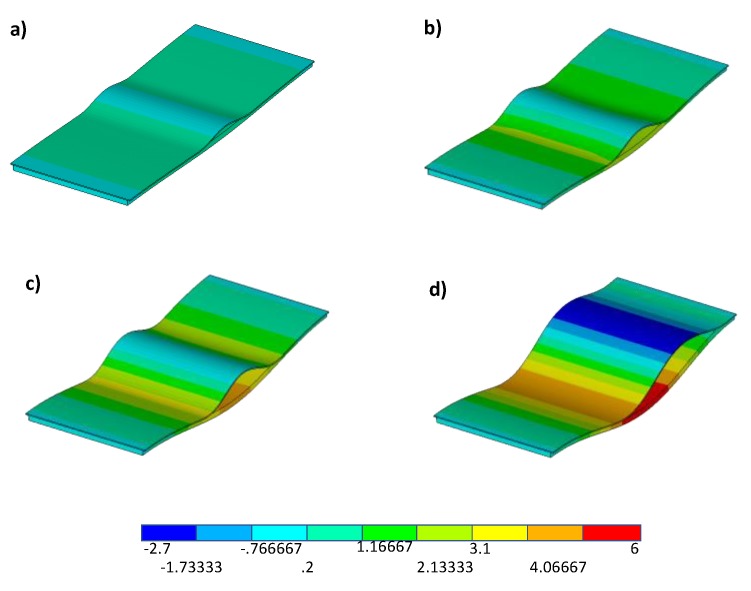
Numerical out-of-plane displacement contour plot for material sensitive to fibre bridging (units in mm) at (**a**) 0.3 mm applied displacement, (**b**) 0.4 mm applied displacement, (**c**) 0.55625 mm applied displacement, and (**d**) 0.73125 mm applied displacement.

Figure 21 and Figure 22 show the numerically deformed shapes with the out-of-plane displacement contour plot, evaluated at four fixed values of the applied displacement, respectively, for the sample characterized by toughened material and the sample characterized by material sensitive to fibre bridging.

Figure 21 and Figure 22 point out the delay in the delamination growth phenomenon and, hence, in the snap-through phenomenon observed for the toughened material configuration, characterised by a constantly higher GIc, with respect to the material sensitive to fibre bridging configuration. Actually, the configuration made of material sensitive to fibre bridging (MFB) is characterised by a lower starting value of the fracture toughness, if compared to the toughened material (TOUGH). This leads to an earlier, even if more stable, delamination growth in the first case (MFB) with respect to the case of toughened material (TOUGH), whose delamination growth initiates with a highly unstable behaviour at higher loads (close to the global buckling load).

**Figure 23:**
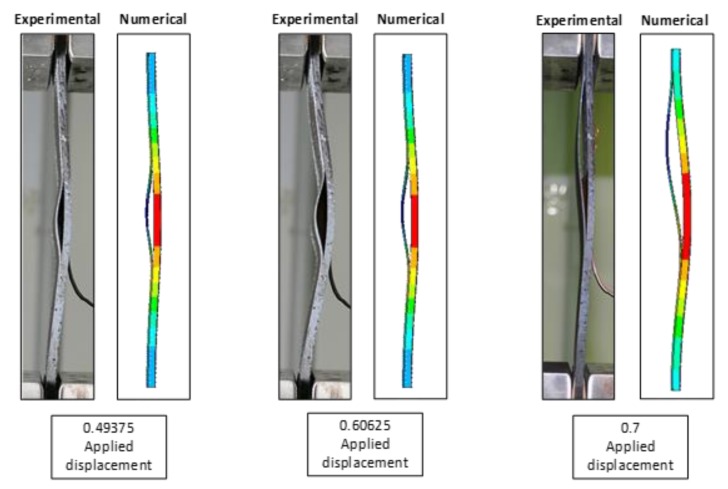
Numerical and experimental deformed shape comparison. Material sensitive to fibre bridging.

Finally, in Figure 23, a comparison between experimental and deformed shapes at fixed values of the applied displacement, for a specimen characterised by sensitivity to fibre bridging, is introduced. The excellent agreement between experimental and numerical delamination shapes demonstrates the capability of the adopted numerical tool in predicting the compressive behaviour of the analysed delaminated composite plates, even in the presence of highly dynamic phenomena, such as the snap-through buckling.

## 5. Conclusions

An experimental/numerical study has been carried out to investigate the interlaminar damage behaviour of delaminated composite plates characterized by different fracture toughness under compressive loading conditions. Two fracture toughness configurations have been analysed: the toughened material configuration, characterised by a constantly high fracture toughness and the configuration sensitive to fibre bridging, characterised by an increasing value of fracture toughness. Actually, an experimental testing campaign was performed to analyse the compressive behaviour of the specimens and the DIC technique was used in order to measure samples displacements and deformations during the tests. Moreover, all the samples were equipped with strain gauges, in different locations, to measure deformations. To numerically simulate the mechanical behaviour of compressed specimen, a numerical tool able to consider the fibre bridging effects was adopted.

The experimental data and the numerical results have been found to be in excellent agreement in terms of deformed shapes, compressive load vs strain curves and out-of-plane displacements.

The two analysed configurations showed almost the same compressive behaviour characterised by the local delamination buckling onset, delamination growth onset, global buckling of the plate and transition from the global buckling shape to the second buckling shape (snap-through buckling phenomenon) caused by a sudden unstable delamination growth.

Differences in terms of delamination growth behaviour have been found, as expected, between the two analysed material configurations. Indeed, the coupons made of material sensitive to fibre bridging are characterised by a lower (standard) value of the fracture toughness at the beginning of the delamination growth phenomenon, which leads to an earlier, even if more stable, delamination growth, if compared to the toughened material configuration, whose delamination growth initiates with a highly unstable behaviour at higher loads (close to the global buckling load). These differences lead to a higher global buckling load for the toughened composite plates and to a delay in the snap-through phenomenon for these plates, if compared to the composite plates sensitive to fibre bridging. Actually, the snap-through phenomenon introduces dynamic effects which cannot be simulated by the tools used for the numerical prediction. Indeed, an underestimation of the experimental ultimate strains has been found; however, a tool has been found able to capture the difference between the two material configurations and the main phenomena occurring during the compression.

## Figures and Tables

**Table 1:** Tests matrix.

	SG1bis	SG1		SG2		DIC
	0°	0°	90°	0°	90°	
TOUGH#1		☑	☑	☑	☑	
TOUGH#2		☑	☑	☑	☑	
TOUGH#3	☑					☑
MFB#1		☑	☑	☑	☑	
MFB#2		☑	☑	☑	☑	
MFB#3	☑					☑

**Table 2:** Summary of experimental results.

	Global Buckling		Delamination Buckling		Snap-Through Buckling	
	Load [kN]	Strain [με]	Load [kN]	Strain [με]	Load [kN]	Strain [με]
TOUGH#1	44.37	−1780	39.4	−1490	40.4	−9960
TOUGH#2	45.2	−1790	39.1	−1480	40.3	−8700
TOUGH#3	45.2	−1785	39.3	−1483	40.3	−8790
MFB#1	41.2	−1600	39.5	−1470	39.3	−6830
MFB#2	40.9	−1600	39.1	−1475	39.0	−6750
MFB#3	40.1	−1590	39.2	−1471	39.4	−7000
